# Optimization of Sample Preparation and Instrumental Parameters for the Rapid Analysis of Drugs of Abuse in Hair samples by MALDI-MS/MS Imaging

**DOI:** 10.1007/s13361-017-1766-0

**Published:** 2017-08-11

**Authors:** Bryn Flinders, Emma Beasley, Ricky M. Verlaan, Eva Cuypers, Simona Francese, Tom Bassindale, Malcolm R. Clench, Ron M. A. Heeren

**Affiliations:** 1 FOM-Institute AMOLF, Science Park 104, 1098 XG Amsterdam, The Netherlands; 2 Center for Mass Spectrometry Imaging, Biomolecular Sciences Research Center, City Campus, Sheffield Hallam University, Howard Street, Sheffield, S1 1WB UK; 3 KU Leuven Toxicology and Pharmacology”, Herestraat 49, PO 922, 3000 Leuven, Belgium; 4 Maastricht Multimodal Molecular Imaging Institute (M4I), University of Maastricht, Universiteitssingel 50, 6229 ER Maastricht, The Netherlands

**Keywords:** MALDI-MSI, Cocaine, Metabolites, Raster imaging

## Abstract

Matrix-assisted laser desorption/ionization-mass spectrometry imaging (MALDI-MSI) has been employed to rapidly screen longitudinally sectioned drug user hair samples for cocaine and its metabolites using continuous raster imaging. Optimization of the spatial resolution and raster speed were performed on intact cocaine contaminated hair samples. The optimized settings (100 × 150 μm at 0.24 mm/s) were subsequently used to examine longitudinally sectioned drug user hair samples. The MALDI-MS/MS images showed the distribution of the most abundant cocaine product ion at *m/z* 182. Using the optimized settings, multiple hair samples obtained from two users were analyzed in approximately 3 h: six times faster than the standard spot-to-spot acquisition method. Quantitation was achieved using longitudinally sectioned control hair samples sprayed with a cocaine dilution series. A multiple reaction monitoring (MRM) experiment was also performed using the ‘dynamic pixel’ imaging method to screen for cocaine and a range of its metabolites, in order to differentiate between contaminated hairs and drug users. Cocaine, benzoylecgonine, and cocaethylene were detectable, in agreement with analyses carried out using the standard LC-MS/MS method.

**Electronic supplementary material:**

The online version of this article (doi:10.1007/s13361-017-1766-0) contains supplementary material, which is available to authorized users.

**Graphical Abstract:**
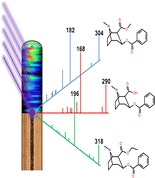
ᅟ

## Introduction

Hair testing is a powerful tool routinely used for the detection of drugs of abuse in toxicology and forensic applications [1–3]. The analysis of hair is highly advantageous as it can provide prolonged detection and chronological information about drug intake or chemical exposure in contrast to the analysis of biological fluids [4]. However, current methodology routinely involves complex and time-consuming homogenization, derivatization, sample-clean up, and extraction techniques followed by gas or liquid chromatography coupled with mass spectrometry (GC-MS or LC-MS). Also these techniques require large amounts of hair sample (10–100 mg) and can only provide the chronological information per month (based on the average growth rate of 1 cm/mo).

Matrix-assisted laser desorption/ionization-mass spectrometry imaging (MALDI-MSI) is well established for the detection and imaging of drugs and pharmaceuticals in tissues. However, it is increasingly being used for the analysis of drugs of abuse in hair, as it offers several advantages over the currently established techniques, such as requiring fewer hair samples, simpler and faster sample preparation, and providing more accurate and visual chronological information in hours or days.

MALDI-MSI has been used to monitor the distribution of a wide range of compounds, including drugs of abuse, pharmaceuticals, and other compounds in single hair samples, such as cocaine [5], methamphetamine [6, 7], ketamine [8], cannabinoids [9], tilidine [10], zolpidem [11, 12], and nicotine [13]. New techniques have also been introduced into the field, such as infrared-matrix-assisted laser desorption electrospray ionization-mass spectrometry imaging (IR-MALDESI-MSI), which has been used to monitor the distribution of the antiretroviral efavirenz in hair samples from HIV infected patients [14]. Recently, mass spectrometry imaging techniques have been used to address some of the current issues with forensic hair testing, such as the process/rate of drug incorporation [15], the effects of cosmetic treatment [16], and the consequences of different washing procedures [17].

Whilst these examples show it is possible to monitor the distribution of a wide range of compounds in single hair samples, multiple hairs need to be analyzed in order to account for the different growth phases of hair. As a result, depending on the length and number of the hair samples or the spatial resolution, it can take several hours to a few days to acquire images with the conventional spot-to-spot acquisition method. One way to overcome this and improve the speed of analysis is to use “raster imaging” mode. This method of data acquisition is achieved by continuously firing the laser in rows across a sample. The generated data is placed into a bin at selected intervals during the raster, which is based upon the selected spatial resolution and sampling speed [18, 19]. Another issue is the extraction efficiency of the embedded drugs by the matrix solution. As the drugs are considered to be bound to melanin inside the core of the hair, it remains difficult to know whether the drug is completely extracted from the hair by the MALDI matrix, especially through the impermeable outer surface; this can be overcome by longitudinally sectioning the hair samples prior to analysis.

In the work reported here, instrumental and experimental parameters were optimized to rapidly generate high quality images of longitudinally sectioned drug user hair samples using continuous raster imaging. In order to quantify the detected drug, a novel method for preparing a calibration line on longitudinally sectioned hair was developed. To further confirm if the detected drugs and metabolites are indicative of actual ingestion, a multiple reaction monitoring (MRM) method was developed to screen for unique metabolites.

## Experimental

### Materials

Alpha-cyano-4-hydroxycinnamic acid (CHCA), cocaine (COC), benzoylecgonine (BZE), norcocaine (NCOC), cocaethylene (CE), ecgonine methyl ester (EME), anhydroecgonine methyl ester (AEME), and dichloromethane (DCM) were purchased from Sigma Aldrich (Schnelldorf, Germany). Acetonitrile (ACN), methanol (MeOH), and trifluroacetic acid (TFA) were purchased from Biosolve (Valkenswaard, The Netherlands).

### Sample Preparation

Hair samples were collected from volunteer drug users and hair samples of non-users were collected from volunteers and analyzed as negative controls. Hair samples were decontaminated using two 10 mL dichloromethane washes for 1 min by shaking. After washing, the hair samples were left dry at room temperature [20, 21]. Longitudinal sections of hair samples were prepared using the previously reported method [22]. Briefly, the hair sample was affixed onto a metal plate that contains grooves ranging from 20 to 80 μm. Whilst holding the other end of the hair sample with a gloved finger, a holder with a blade fixed at a 20° angle was run along the length of the hair. After visual inspection using a Leica DM RX light microscope (Leica, Wetzlar, Germany) equipped with a Nikon DM100 digital camera (Nikon, Tokyo, Japan), the hair samples were mounted onto a glass slide using double-sided tape. Control hair samples were placed into a 1 mg/mL solution of cocaine (50:50 acetonitrile:water) before mounting onto a glass slide using double sided tape.

### Preparation of Standards for Quantitation

Cocaine standards were prepared from a 1 mg/mL stock solution to give the following standards: 0.1, 0.2, 0.5, 1, 2, 5, and 10 ng/μL in 70% acetonitrile. In order to achieve a homogenous and uniform deposition, the cocaine standards were sprayed onto longitudinal sectioned control hair samples using the Suncollect automated pneumatic sprayer (Sunchrom, Friedrichsdorf, Germany) with the aid of stencils made from polylactic acid. The stencils (containing square holes that are 2 mm^2^) were made using a Ultimaker Original 3D printer (Ultimaker, Geldermalsen, The Netherlands). The standards were sprayed in a series of 30 layers. The initial layer was sprayed at 10 μL/min, then stepped up from 20 μL/min to 30 μL/min, and subsequent layers were sprayed at 40 μL/min. The hair samples were mounted onto a glass slide using double sided tape.

### Matrix Application

The samples were coated with 7 mg/mL CHCA in 50:50 acetonitrile:water with 0.2% TFA using a Bruker ImagePrep (Bruker Daltonics, Bremen, Germany).

### Instrumentation

All data were acquired in positive ion mode on an Applied Biosystems/MDS Sciex hybrid quadrupole time-of-flight mass spectrometer (Q-Star Pulsar-*i*) with an orthogonal MALDI ion source (Applied Biosystems, Foster City, CA, USA) and a neodymium-doped yttrium aluminium garnet (Nd:YAG) laser (355 nm, 1 KHz). The laser power was 30 (1000 Hz, 3.2 μJ) and the laser beam had an elliptical spot size of 100 × 150 μm. Image acquisition was performed using the “raster image” mode [18, 23]. Images were generated using the freely available Novartis Biomap 3.8.0.4 software (www.maldi-msi.org). MALDI-MS spectra were obtained in positive ion mode in the mass range between *m/z* 50 and 1000. Declustering potential 2 was set at 15 arbitrary units and the focus potential at 10 arbitrary units, with an accumulation time of 0.999 s. The MALDI-MS/MS spectra were obtained using argon as the collision gas; the declustering potential 2 was set at 15 and the focusing potential at 20, and the collision energy and collision gas pressure were set at 20 and 5 arbitrary units, respectively.

Dynamic pixel imaging was employed to perform MRM imaging experiments. The method was optimized using standards of cocaine and its metabolites (100 ng/μL in 70% methanol), and the most abundant product ions were selected for imaging. The laser power was 80% (1000 Hz, 8 μJ), the instrument parameters were accumulation time of 0.4 s, seconds/spot 2.4 s, and the mass range was ±2 u for each product ion. Images were generated using the oMALDI server 5.1 software (MDS Sciex, Concord, ON, Canada).

### Data Processing

For presentation purposes, mass spectra from the Analyst QS 1.1 software were exported in the form of text files and imported into mMass software, an open-source mass spectrometry software used for mass spectral processing [24].

## Results and Discussion

In the initial phase of the study, the optimization of instrumental parameters was carried out:

### Optimization of Spatial Resolution and Raster Speed for MALDI-MS/MS Imaging

**Figure 1:**
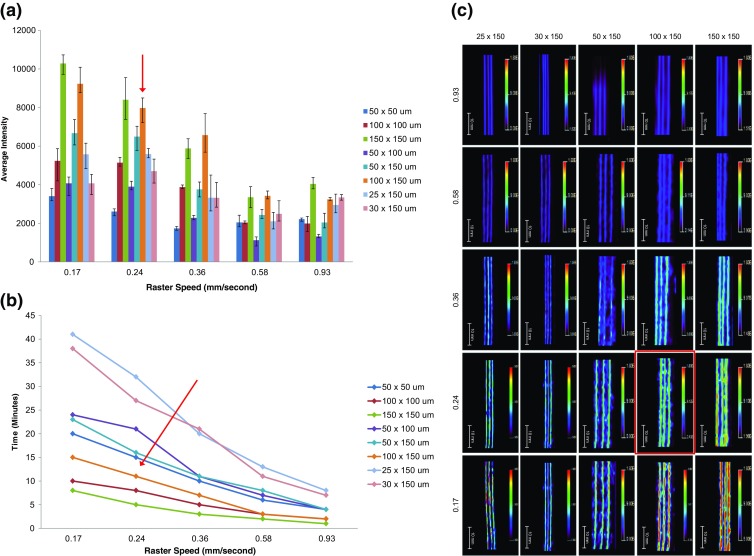
Graphs to determine the optimal spatial resolution and raster speed for imaging the distribution of cocaine in hair samples by MALDI-MS/MS imaging. The graphs show (**a**) the average intensity of the cocaine product ion at *m/z* 182 at each spatial resolution tested at different raster speeds, and (**b**) the time taken for each spatial resolution tested at different raster speeds. (**c**) MALDI-MS/MS images of cocaine contaminated hair samples analyzed used to determine the optimal parameters showing the distribution of the product ion at *m/z* 182. The highlighted image and areas indicated by the red arrows show the determined optimal parameters

To determine the optimal spatial resolution and raster speed intact cocaine contaminated hair samples were analyzed in triplicate, and these results were plotted as a function of the average intensity and time, respectively. The results from these experiments are shown in Figure 1.

The average intensity of the product ion of cocaine at *m/z* 182 for each of the spiked hair samples analyzed (n = 3) was determined using the region of interest (ROI) tool in the Biomap 3.8.0.4 imaging software. The results shown in Figure 1 show that analysis of samples at a high spatial resolution results in a decreased sample throughput and sensitivity. This is due to the increased number of rasters and extensive oversampling. Conversely, analysis of samples at a lower spatial resolution results in an increased sample throughput and sensitivity, because of the reduced number of rasters and a fresh area being consistently sampled.

However, it should be noted that when performing the analysis of hair samples at a lower spatial resolution, the results from individual hairs begin to merge. This is observed in the MALDI-MS/MS images (Figure 1c), therefore when preparing hair samples the spacing between the hair samples needs to be taken into account. Whilst high spatial resolution imaging is possible, it may not be necessary, especially across the width of the hair, as the chronological information is obtained longitudinally along the length of the hair. In addition, the incorporation rate and keratinization of drug into the hair can take several days.

Based on the findings of this study, the optimal spatial resolution was determined to be 100 × 150 μm and the optimal raster speed was 0.24 mm/s (416 shots/pixel). Whilst it may appear that imaging the hair samples at 150 × 150 μm, 0.17 mm/s is optimal, the corresponding image shows the hairs begin to merge into one; in addition, there is not much gain in intensity. As the spatial resolution along the length of the hair is 150 μm, each pixel is equivalent to around 12 h of growth. This allows for a much narrower time frame of detection than the standard GC-MS and LC-MS methods, which can only provide information about drug use averaged over a 1 mo period.

### Determination of Optimal Sample Orientation

**Figure 2:**
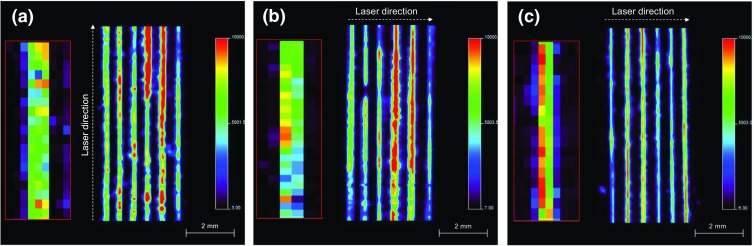
MALDI-MS/MS images of cocaine contaminated hair samples analyzed in different orientations, showing the distribution of the product ion at *m/z* 182. (**a**) Horizontal direction (150 × 100 μm), (**b**) vertical direction (150 × 100 μm), and (**c**) vertical direction (100 × 150 μm). The inserts show the number of pixels per hair

In order to determine if the orientation of the hair samples in relation to the movement of the laser affects the results, six cocaine contaminated hair samples were analyzed in different orientations using the optimized settings. The MALDI-MS/MS images of the cocaine contaminated hair samples are shown in Figure 2.

The MALDI-MS/MS images show cocaine contaminated hair samples analyzed in both the horizontal (Figure 2a) and vertical (Figure 2b and c) orientations. The images show that using the optimized settings clearly differentiates between individual hairs.

The MALDI-MS/MS image shown in Figure 2a shows hair samples analyzed in the horizontal orientation, and the insert shows each hair consists of around to 3–4 pixels. Whereas the image in Figure 2b shows hair samples analyzed in the vertical orientation, the insert shows that each hair consists of 2–3 pixels. The MALDI-MS/MS image in Figure 2c shows better separation, which could be due to the elliptical laser spot size (100 × 150 μm). This is also observed in the insert that shows an expanded view of a single hair prior to smoothing, which consists of around to 2–3 pixels per hair. This experiment shows that hair samples can be analyzed in either orientation; however, the spatial resolution needs to be adjusted accordingly. For subsequent experiments, the hair samples were analyzed in the horizontal orientation with the laser running parallel (150 × 100 μm).

### MALDI-MS/MS Imaging of Longitudinal Sectioned Drug User Hair Samples

**Figure 3:**
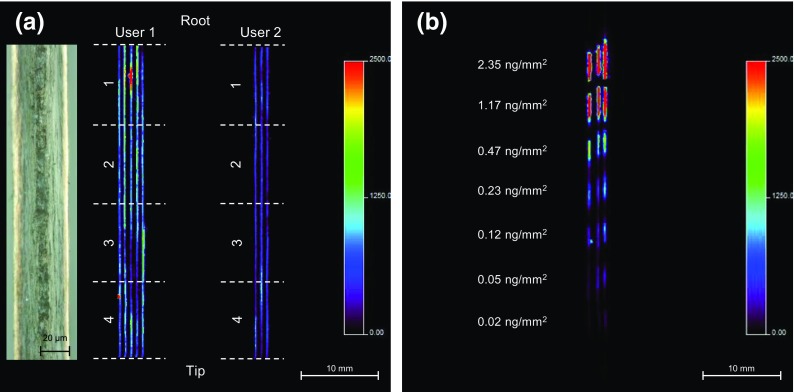
MALDI-MS/MS images of (**a**) longitudinally sectioned drug user hair samples (insert shows optical image of longitudinally sectioned hair), and (**b**) longitudinally sectioned control hair samples sprayed with a cocaine dilution series. The MALDI-MS/MS image shows the distribution of the product ion at *m/z* 182, derived from the precursor ion of cocaine at *m/z* 304

Once the spatial resolution and raster speed was optimized to produce the best quality image in the shortest time, the method was applied to monitor the distribution of cocaine in a number of longitudinally sectioned hair samples from cocaine users. In order to quantify the amount of cocaine present in the hair samples, control hair samples sprayed with a cocaine dilution series were also analyzed. The MALDI-MS/MS images of the cocaine user hair samples and cocaine standard hair samples are shown in Figure 3.

The MALDI-MS/MS image (Figure 3a) shows the distribution of the most abundant cocaine product ion at *m/z* 182, which is formed by the neutral loss of benzoic acid from the intact molecule and was detected in both user hair samples. In contrast, it was not detected in the longitudinal sectioned control hair sample; due to the number of hair samples available from the second user, only three hairs were analyzed. The length of the analyzed hair samples was 4 cm; given the average growth rate of human hair is approximately 1 cm per month, this corresponds to a growth period of 4 mo [25]. Since the spatial resolution along the hair is 150 μm, each pixel is equivalent to around 12 h of growth. The analysis of the longitudinally sectioned user hair samples took 3 h and 22 min (136 s per raster). This is around six times faster in comparison to the standard spot-to-spot acquisition method at this spatial resolution, which takes around 18 h. Analysis with the current methodology takes around 1 h; however, the sample preparation takes approximately 1 d. In contrast, the sample preparation for MALDI-MSI takes around 1 h; along with the optimized settings it takes approximately 4 h to perform the entire experiment. This is six times faster than the currently established method. The insert shows a close-up view of a longitudinally sectioned hair sample, prepared using the previously published method [22]. The image clearly shows minimal damage to the hair with the medulla in the center surrounded by the cortex and the cuticle on the edge of the hair.

In order to quantify the amount of cocaine in the longitudinally sectioned user hair samples, a cocaine dilution series was prepared. This was initially spotted onto the glass slide next to the hair samples; however, this resulted in an uncontrollable deposition due to spreading. Therefore, to overcome this issue, the cocaine dilution series was sprayed onto longitudinally sectioned control hair samples using the described method, in order to reproducibly produce uniform and homogenous standards as shown in Figure 3b. The obtained image does suggest that this method of standard deposition has resulted in homogenous and uniform deposition. A decreasing response with respect to the concentration is clearly observable with good reproducibility for each hair sample (see Supplementary Figure S1). The concentration per standard was reported in ng/mm^2^, which was calculated from the parameters used to spray the cocaine standards. The analysis of the quantitation hair standards took 1 h and 10 min. It should be noted that the control hair samples used for the calibration curve were not the same color as those from the drug users, and other information such as race and gender was not available. Ideally the hair samples used for quantitation should be matched based on hair color, race, and gender.

Using the ROI tool of the Biomap 3.8.0.4 software, the average intensity of the calibration standards (Supplementary Figure S1) and the four segments from both of the user hairs (Supplementary Figure S2) were determined. The calibration curve was linear over two orders of magnitude (R^2^ = 0.9908). Using the calibration curve the concentration of cocaine per segment for the first user was determined to be 0.437, 0.389, 0.340, and 0.305 ng/mm^2^ (1–4), whereas the concentration of cocaine per segment for the second user was determined to be 0.151, 0.154, 0.1720, and 0.186 ng/mm^2^ (1–4). These results indicate both users have a prolonged history of cocaine use and that the first user is a heavier user in contrast to the second user; this is also apparent in in the MALDI-MS/MS image.

### MALDI-MS/MS Imaging of Cocaine Metabolites in Drug User Hair Samples

One way to determine if a detected drug is present due to ingestion rather than environmental contamination is to monitor the presence of unique metabolites [20, 26]. In the case of cocaine, cocaethylene (a metabolite formed by the simultaneous consumption of cocaine and ethanol), norcocaine (an in-vivo metabolite of cocaine), and anhydroecgonine methyl ester (a pyrolysis product formed when crack cocaine is smoked). Other metabolites, such as benzoylecgonine, the main metabolite of cocaine, can also be formed by environmental degradation [27]. In order to screen the drug user hair samples for cocaine and its metabolites, a MRM imaging method using dynamic pixel imaging was developed. Dynamic pixel imaging is a technique that enables multiple experiments to be performed consecutively in a single acquisition. This is because the target plate is moved around within each pixel, which enables longer acquisition time per pixel and thus multiple experiments to be performed [28]. The transitions for cocaine and its metabolites were as follows: cocaine (*m/z* 304.15→182.12), cocaethylene (*m/z* 318.17→196.15), norcocaine (*m/z* 290.13→136.09), benzoylecgonine (*m/z* 290.15→168.11), ecgonine methyl ester (*m/z* 200.16→182.13), and anhydroecgonine methyl ester (*m/z* 182.13→118.06). The MALDI-MS/MS spectra of cocaine and its metabolites are shown in Supplementary Figure S3.

**Figure 4:**
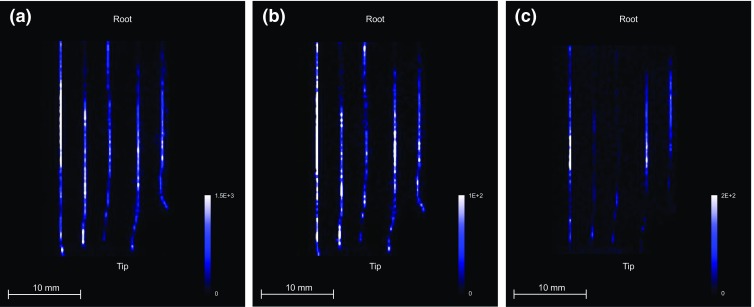
MALDI-MS/MS images of longitudinally sectioned drug user hair samples. The images show the distribution of (**a**) cocaine (*m/z* 304.15→182.12), (**b**) benzoylecgonine (*m/z* 290.15→168.11), and (**c**) cocaethylene (*m/z* 318.17→196.15)

A requirement for the use of the dynamic pixel imaging method is that the hairs needed to be spaced as far apart as possible in order to distinguish individual hairs. This is due to the figure eight movement of the sample stage during the acquisition, and as a result the best spatial resolution that could be achieved was 250 × 250 μm. The MALDI-MS/MS images of cocaine and its metabolites in the longitudinal sectioned hair samples acquired in this manner are shown in Figure 4.

The length of the analyzed hair samples was 3 cm, and given the average growth rate of human hair is around 1 cm per month, this corresponds to a growth period of 3 mo.. Since the spatial resolution along the hair is 250 μm, this corresponds to 18 h of growth. Cocaine, the major metabolite benzoylecgonine, and the metabolite cocaethylene were detected in the drug user hair sample. This was also confirmed using the routine LC-MS/MS analysis method.

## Conclusions

The use of MALDI-MS/MS imaging for the rapid screening of drugs of abuse in hair samples using continuous raster imaging has been presented. Optimization of instrumental and experimental parameters such as the spatial resolution, raster speed, and sample orientations were performed in order to rapidly analyze hair samples without compromising the quality of the images. Whilst these settings are specific to this instrument, they provide a starting point for the optimization of these parameters on other instruments operating in raster imaging mode. Using the optimized settings (100 × 150 μm at 0.24 mm/s), the analysis of the longitudinally sectioned hair samples of two drug users took approximately 3 h, which is six times faster in comparison with the standard spot-to-spot acquisition method at this spatial resolution, which takes around 18 h. In order to quantify the amounts of cocaine in longitudinally sectioned drug user hair samples, a novel method for the preparation of standards was developed. In order to determine if the detected drugs present are from actual abuse rather than external contamination, a MRM imaging method utilizing ‘dynamic pixel’ imaging in combination with longitudinally sectioned hair was developed. By screening for unique cocaine metabolites that can only be formed in vivo, the confirmation of ingestion of cocaine could be ascertained. Cocaine, benzoylecgonine, and cocaethylene were present, which was consistent with the standard LC-MS/MS method. The work presented here also shows that if required, faster analysis is possible but the spatial resolution and spacing between hair samples needs to be adjusted accordingly.

## Electronic supplementary material

Below is the link to the electronic supplementary material.

ESM 1 (DOCX 3401 kb)
