# Supplementary material for: Optimization of Sample Preparation and Instrumental Parameters for the Rapid Analysis of Drugs of Abuse in Hair samples by MALDI-MS/MS Imaging
Source: J Am Soc Mass Spectrom. 2017 Aug 11;28(11):2462–8. doi: 10.1007/s13361-017-1766-0 (PMC5645433; doi:10.1007/s13361-017-1766-0)
Supplement: Supplementary file 1 — (DOCX 3401 kb) [file 13361_2017_1766_MOESM1_ESM.docx]

**Optimization of sample preparation and instrumental parameters for the rapid analysis of drugs of abuse in hair samples by MALDI-MS/MS imaging.**

Bryn Flinders ^1,4^, Emma Beasley^2^, Ricky M. Verlaan^4^, Eva Cuypers ^3^, Simona Francese^2^, Tom Bassindale^2^, Malcolm R. Clench^2^, Ron M. A. Heeren ^1,4*^

**Supplemental Figures**

**Supplemental Figure 1:** Calibration curve generated from the cocaine dilution series sprayed onto longitudinally section control hair samples (n=3).

**Supplemental Figure 2:** Graph showing the average intensity of the four segments from the two users obtained from ROIs over the hairs analyzed.

**Supplemental Figure 3:** MALDI-MS/MS spectra of cocaine and its metabolites. A) cocaine (*m/z* 304.15→182.12), B) cocaethylene (*m/z* 318.17→196.15), C) benzoylecgonine (m/z 290.15→168.11), D) ecgonine methyl ester (*m/z* 200.16→182.13), E) norcocaine (*m/z* 290.13→136.09) and F) anhydroecgonine methyl ester (*m/z* 182.13→118.06).
